# Genome-Wide Characterization and Expression Pattern Analysis Insights into Plant *NBS-LRR* Gene Family of *Salvia miltiorrhiza*

**DOI:** 10.3390/ijms26189063

**Published:** 2025-09-17

**Authors:** Linglong Luo, Jian Wang, Guanghong Cui, Jinfu Tang, Ying Ma, Baolong Jin, Ping Su, Yifeng Zhang, Yanan Wang, Tong Chen, Juan Guo, Luqi Huang

**Affiliations:** 1 State Key Laboratory for Quality Ensurance and Sustainable Use of Dao-di Herbs, National Resource Center for Chinese Materia Medica, China Academy of Chinese Medical Sciences, Beijing 100700, China; linglongluo@163.com (L.L.); jianwang2021@126.com (J.W.); guanghongcui@163.com (G.C.); jinfutang@126.com (J.T.); xiaoma1110@126.com (Y.M.); jblhandan@163.com (B.J.); suping120@163.com (P.S.); yifengzhang06@126.com (Y.Z.); wangyn@nrc.ac.cn (Y.W.); chentong_biology@163.com (T.C.); 2 Dalian Institute of Marine Traditional Chinese Medicine, Dalian 116000, China

**Keywords:** *NBS-LRR* genes, plant disease resistance, *Salvia miltiorrhiza*, genome wide analysis, secondary metabolism

## Abstract

The *NBS-LRR* genes constitute the largest class of resistance (R) proteins in plants, capable of recognizing pathogen-secreted effectors to trigger immune responses. However, systematic studies of *NBS-LRR* genes in medicinal plants have not yet been reported. In this study, we performed a comprehensive genome-wide identification and analysis of the *NBS-LRR* gene family in the medicinal plant *Salvia miltiorrhiza*. A total of 196 *NBS-LRR* genes were identified, among which 62 possessed complete N-terminal and LRR domains. Multiple NBS-LRR proteins extracted from other model plants can be classified and distinguished on the phylogenetic tree according to subfamilies CNL, TNL, and RNL. Comparative analysis revealed a marked reduction in the number of TNL and RNL subfamily members within the Salvia species. Analysis of the expression patterns of *SmNBS-LRR* genes with transcriptomes data revealed a close association between *SmNBS-LRRs* and secondary metabolism. Promoter analysis demonstrated an abundance of *cis*-acting elements in *SmNBS* genes related to plant hormones and abiotic stress. Our study enhances the understanding of the *NBS-LRR* gene family in medicinal plants and provides a foundation for future functional characterization of the *NBS-LRR* genes in *S. miltiorrhiza* and its potential application in disease-resistance breeding.

## 1. Introduction

Danshen (*Salvia miltiorrhiza* Bge.) is a well-known medicinal plant of the genus Salvia in the family Lamiaceae. Its roots contain bioactive compounds such as tanshinones and phenolic acids, which are widely used in the treatment of cardiovascular and cerebrovascular diseases [1,2], as well as for their anti-inflammatory and antibacterial properties [3]. However, as a perennial species, the incidence of diseases in *S. miltiorrhiza* increases with extended cultivation, leading to leaf spot, viral infections, and root rot [4], which are among the major factors responsible for reduced yield and deteriorated quality. At a global scale, yield losses in crops due to pathogens and pests are estimated at 10% to 41.1% [5].

Plants have evolved sophisticated defense strategies, most prominently a two-layered immune system that enables recognition and elimination of pathogens [6,7]. The first layer, pathogen-associated molecular pattern (PAMP)-triggered immunity (PTI) [8], is initiated when cell surface-localized receptors recognize conserved pathogen signatures. The second layer, termed effector-triggered immunity (ETI) [9], is mediated by intracellular resistance (R) proteins that detect effector proteins delivered by pathogens into plant cells, thereby activating a stronger immune response, often accompanied by hypersensitive response (HR) and programmed cell death (PCD). Recent studies have revealed that PTI and ETI can act synergistically to enhance plant immune responses, rather than functioning as independent pathways [10]. R genes activate ETI in plants by recognizing avirulence (Avr) proteins secreted by pathogens [11]. Notably, approximately 80% of functionally characterized R genes belong to the *NBS-LRR* (or *NLR*) gene family [12,13,14], which contains a conserved nucleotide-binding site (NBS) domain and a C-terminal leucine-rich repeat (LRR) domain, thereby making it a major component of the plant immune system [15].

The nucleotide-binding site (NBS) conserved domain is the hallmark of *NLR* genes and functions by binding and hydrolyzing ATP to activate downstream immune signaling [16]. And Leucine-Rich Repeat (LRR) domains [17,18] are responsible for recognizing diverse effectors released by pathogens [19]. In 1994, scientists cloned the first plant NBS-LRR protein, RPS2, from *Arabidopsis thaliana* [20], this protein mediates resistance against *Pseudomonas syringae* by recognizing the effector protein AvrRpt2. The *Oryza sativa* CNL subfamily protein Pita directly recognizes the effector AVR-Pita of the rice blast fungus through its LRR domain, thereby activating immune signaling pathways [21], and also plays an important role in dimerization and signal transduction [22]. In *A. thaliana*, the LRR receptor protein RLP23 associates with the lipase-like proteins EDS1 and PAD4, as well as the ADR1 protein [23], forming a supramolecular complex that serves as a convergence point for defense signaling cascades, thereby conferring immunity against pathogens.

To date, most research on the *NBS-LRR* gene family has primarily concentrated on model plants and economically important crops, while studies in medicinal plants remain largely unreported. This knowledge gap is particularly striking for *S. miltiorrhiza*, which, despite its importance as a model species for medicinal studies, has not yet been investigated for NBS-LRR protein functions. This significantly limits our understanding of disease resistance mechanisms in *S. miltiorrhiza*. The first inbred line (bh-27) genome of *S. miltiorrhiza* (published in 2020) [24] and the subsequently published genome assemblies [25,26] are a valuable genomic resource for in-depth studies. In this study, we conducted a genome-wide identification and structural analysis of the *NBS-LRR* gene family members in *S. miltiorrhiza*. By integrating stress-induced and hormone-related transcriptome data, we systematically investigated the critical roles of *NBS-LRR* genes in *S. miltiorrhiza* immunity. This work not only fills a major research gap in the molecular biology of medicinal plants but also provides new insights into the immune regulatory mechanisms of *S. miltiorrhiza*.

## 2. Results

### 2.1. Structural and Phylogenetic Characterization of SmNBS-LRR Resistance Genes

Based on domain integrity, plant NBS-LRR proteins can be classified into typical and atypical types. Among the typical NBS-LRRs, classification further depends on the N-terminal domain type, which are divided into three types: toll/interleukin-1 receptor (TIR), coiled-coil (CC), and resistance to powdery mildew 8 (RPW8). The N-terminal or LRR domains of NBS-LRR proteins are often absent, and such proteins are classified as atypical NBS-LRRs. Based on the specific domain deletions, atypical NBS-LRRs can be further categorized into subtypes such as N (NBS only), TN (TIR-NBS), CN (CC-NBS), and NL (NBS-LRR). Different types of NBS-LRR proteins are closely associated with their functions in plants, with both CNL and TNL proteins serving as intracellular receptors in effector-triggered immunity (ETI). Functional studies of NBS proteins have been conducted in model plants; for example, the *A. thaliana*, *Nicotiana tabacum*, *O. sativa*, and *Solanum tuberosum* genomes contain 207, 156, 505, and 447 NBS-LRR proteins, respectively [27,28].

In this study, we analyzed NBS-LRR proteins in the genome and transcriptome data of the model medicinal plant *S. miltiorrhiza*. Using Hidden Markov Model (HMM) profiles obtained from InterPro, we searched the *S. miltiorrhiza* genome and identified 196 genes containing the NBS domain (Figure S1), accounting for 0.42% of all annotated protein-coding genes. Among them, 62 were predicted to be typical NLRs protein with a complete N-terminal domain and an LRR domain. The domain analysis revealed that, among the NLRs in *S. miltiorrhiza*, 2 proteins contain the toll/interleukin-1 receptor (TIR) domain, 75 contain the coiled-coil (CC) domain, and only 1 protein contains the resistance to powdery mildew 8 (RPW8) domain. Phylogenetic and structural analyses indicated that *S. miltiorrhiza* possesses 61 CNLs, and only 1 RNL protein.

**Figure 1:**
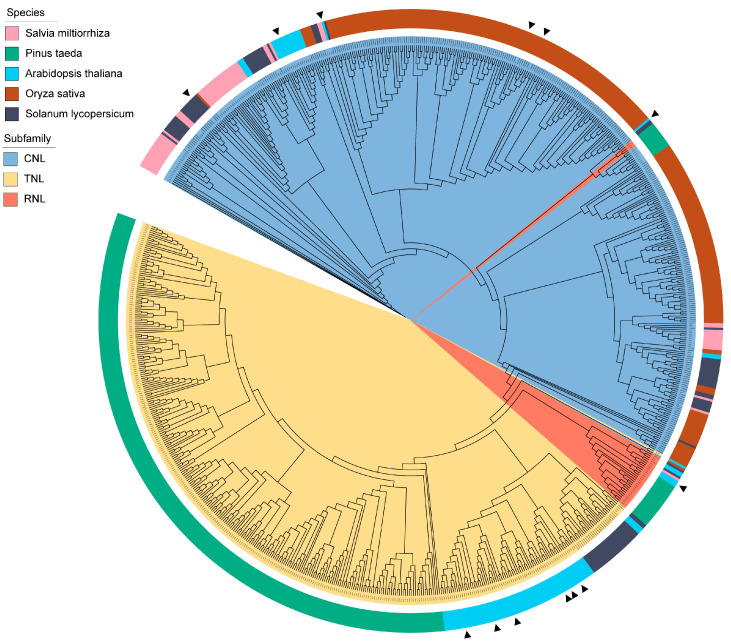
Phylogenetic tree of typical NBS-LRR proteins with complete N-terminal and LRR domains from five species, including *S. miltiorrhiza*, *A. thaliana*, *O. sativa*, *S. tuberosum*, *P. taeda*. In the phylogenetic tree, each different subfamily is highlighted in distinct colors. The outer ring is annotated with species information, and black triangles are used to indicate resistance protein previously reported in the literature, facilitating comparison with functional studies.

By retrieving NBS-LRR proteins containing both complete N-terminal and LRR domains from the genome data of other species and integrating them with the NLRs in *S. miltiorrhiza*, we performed a phylogenetic analysis. A phylogenetic tree was subsequently constructed, comprising a total of 867 NLRs from five species (Figure 1), including *S. miltiorrhiza*, *A. thaliana*, *S. tuberosum*, *Pinus taeda*, and *O. sativa* (101 NLRs from *A. thaliana*, 118 from *S. tuberosum*, 311 from *P. taeda*, and 275 from *O. sativa*). The phylogenetic analysis revealed that all proteins clustered into three branches: CNL, TNL, and RNL. All CNL subfamily from *S. miltiorrhiza* grouped within the same clade as CNL from model plants, while RNL subfamily from all species clustered together into a single branch. Among them, SmNBS35/49/51 clustered in the same branch as the *A. thaliana* resistance protein RPH8A [29], which is capable of inducing a hypersensitive response in cells. Moreover, SmNBS55 and SmNBS56 clustered with the well-characterized *A. thaliana* resistance protein RPM1, which confers resistance to *Pseudomonas syringae*, suggesting that they may play similar roles in pathogen recognition and immune signaling [30]. Among the proteins that clustered with the reported tomato resistance protein Tm-2, SmNBS83 was identified, which confers strong resistance to tobacco mosaic virus (TMV) in tomatoes by recognizing the Avr viral movement protein (MP) [31]. In the RNL clade, SmNBS167 clustered with the Arabidopsis ADR1 protein [23].

**Figure 2:**
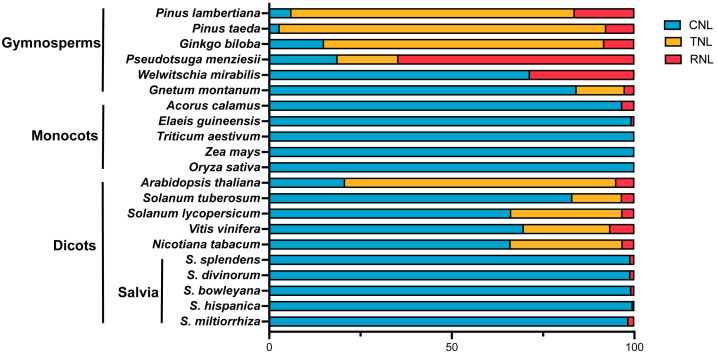
Distribution of typical *NBS-LRR* subfamily members (CNL, TNL, RNL) across multiple species, including five Salvia species, *S. miltiorrhiza*, *S. divinorum*, *S. bowleyana*, *S. hispanica* and *S. splendens*, as well as gymnosperms, monocots, and other dicotyledonous plants.

Analysis of the expansion and contraction of NLR proteins in *S. miltiorrhiza* revealed that, among the 62 identified NLRs, 61 belong to the CNL subfamily and 1 belongs to the RNL subfamily; these findings indicated a notable degeneration of the TNL and RNL subfamilies in *S. miltiorrhiza* at the phylogenetic branch level. The proportion of NLR subfamily proteins varies markedly among plant species (Figure 2). For example, in gymnosperms such as *P. taeda*, the TNL subfamily has undergone significant expansion, comprising 89.3% of the typical *NBS-LRR*s. In contrast, typical TNL and RNL subfamily have been completely lost in monocotyledonous species such as *O. sativa*, *Triticum aestivum*, and *Zea mays*. Comparative analysis with four other Salvia species (*S. miltiorrhiza*, *S. bowleyana*, *S. divinorum*, *S. hispanica*, and *S. splendens*) revealed that none of them contain TNL subfamily, and the number of RNL subfamily member was limited to only one or two copies, which was far fewer than in other angiosperms, such as *A. thaliana*, *N. tabacum*, and *Vitis vinifera* (Figure 2). These findings indicated a clear degeneration of the TNL and RNL subfamilies within the genus Salvia, accompanied by a substantial expansion of the CNL subfamily during Salvia evolution. These results suggested that the *NBS-LRR* resistance genes in *S. miltiorrhiza* may have undergone specialization or homogenization under specific natural selection pressures, leading to distinctive evolutionary divergence and potential functional specialization.

### 2.2. Gene Structure and Cis-Regulatory Element Analysis of SmNBS-LRRs

**Figure 3:**
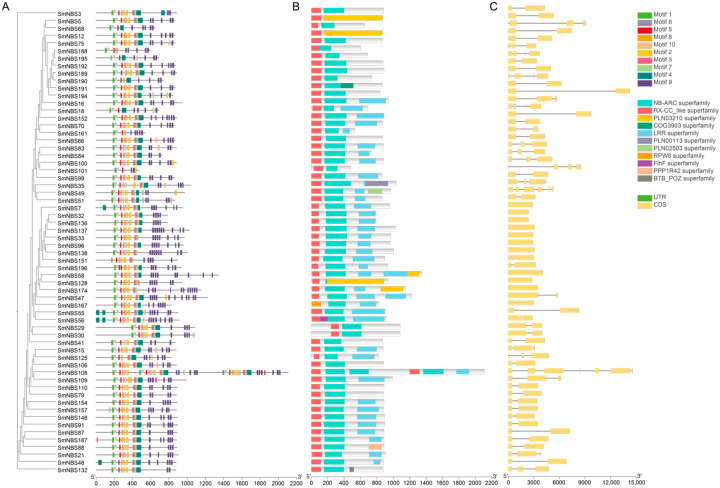
Phylogenetic relationships of 62 typical *SmNBS-LRRs*. (**A**) Conserved motifs; (**B**) conserved domain; (**C**) exons and intron.

MEME analysis identified 10 conserved motifs within the SmNBS-LRR proteins (Figure 3). Most proteins contained these conserved motifs arranged in a consistent order, indicating a high degree of conservation and suggesting that they likely constitute the core components of the NBS-LRR domain in resistance genes (Figure 3A,B). On the other hand, the number of exons in *SmNBS* genes is positively correlated with gene length, and most *S. miltiorrhiza NBS-LRR* genes contain only one or two exons (Figure 3C). This compact gene structure may facilitate rapid transcriptional regulation under stress [32], enabling quicker adaptation to environmental pressures. For example, the SmPR-1 protein family, which is associated with pathogenic response of *S. miltiorrhiza*, is intronless [33]; this conserved single-exon structure highlights both evolutionary conservation and potential functional significance.

**Figure 4:**
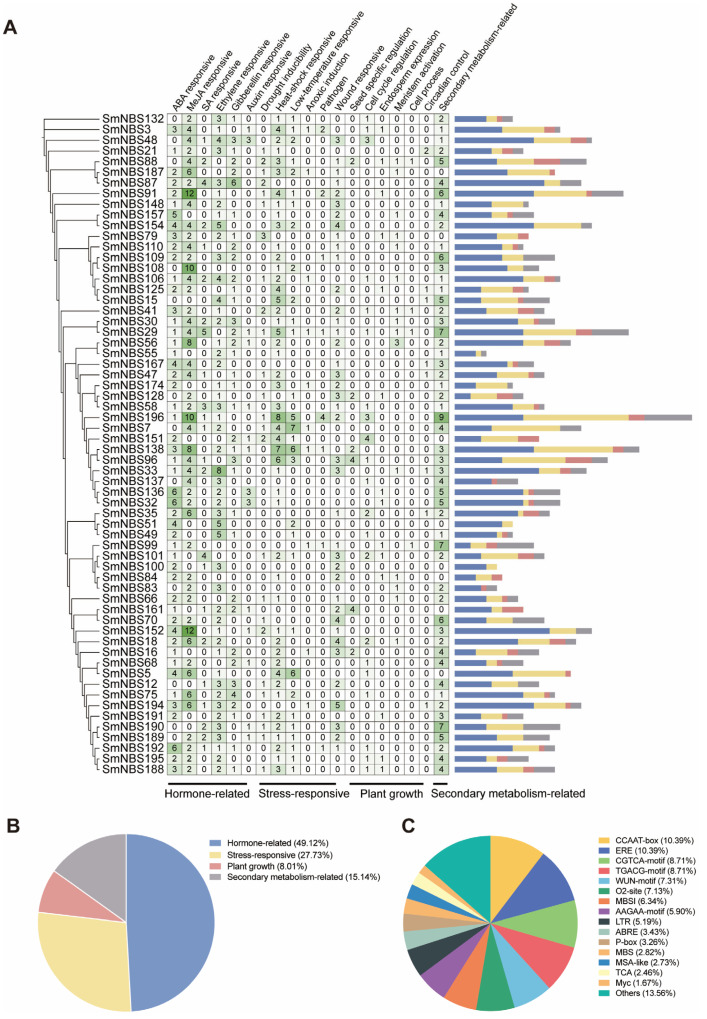
Quantitative statistics of functions of *cis*-regulatory elements of *SmNBS-LRRs*. (**A**) Detailed information on the types and numbers of *cis*-regulatory elements; (**B**) proportional distribution of *cis*-elements across four functional categories; (**C**) proportions of different individual *cis*-element types.

Using the PlantCARE tool, we performed motif analysis on the 2000 bp promoter regions upstream of the start codon of *SmNBS-LRR* genes. The promoter regions contain various cis-regulatory elements (CREs), which were classified and summarized according to their known functions in plants. The results for 62 typical *NBS-LRR* family genes are presented in Figure 4. Based on their functions, CREs were categorized into four groups: hormone-related elements (49.1%), stress-responsive elements (27.7%), plant growth-related elements (8.0%), and secondary metabolism-related elements (15.1%) (Figure 4A,B). Various hormone-responsive elements were detected, including those responsive to salicylic acid (SA), gibberellin (GA), methyl jasmonate (MeJA), and abscisic acid (ABA). Notably, MeJA-related CREs were particularly abundant in certain SmNBS-LRR genes; for example, the promoter region of the differentially expressed gene SmNBS152 contains 12 MeJA-responsive elements, suggesting these genes may play important roles in plant defense mechanisms. Various stress-responsive elements were also identified, including those related to heat shock, low temperature, and wound responses, along with a smaller number of elements associated with plant growth. These results indicate that the promoter regions of typical *NBS-LRR* genes in *S. miltiorrhiza* are enriched with hormone-related and stress-responsive *cis*-regulatory elements.

### 2.3. Expression Patterns and Structural Analysis of SmNBS-LRR Genes Across Different Phylogenetic Clades

**Figure 5:**
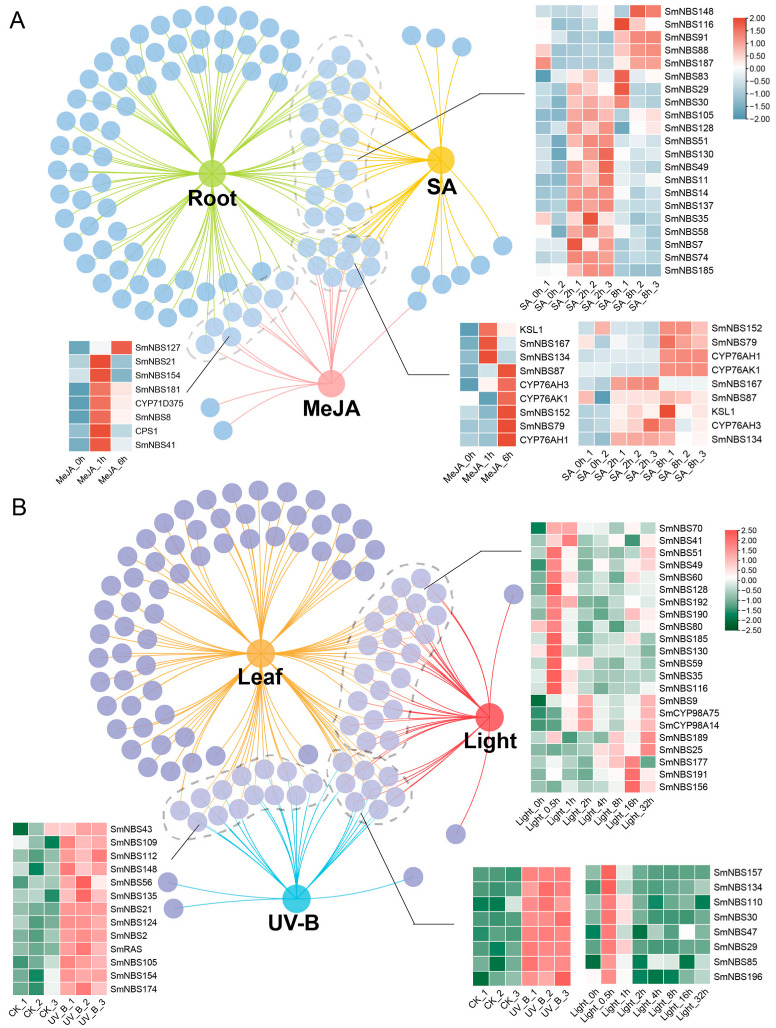
The expression analysis of transcripts of SmNBS-LRR. (**A**) Network Venn diagram of gene expression patterns in roots and after MeJA and SA treatments; (**B**) gene expression patterns in leaf and after UV-B and high light stress treatments.

*S. miltiorrhiza* is regarded as a model medicinal plant due to its rich content of bioactive secondary metabolites, such as tanshinones and phenolic acids. Studies have shown that pathogen-induced stress can activate plant secondary metabolic pathways and significantly promote the accumulation of tanshinones. As core components of plant defense, *NBS-LRR* resistance genes may play regulatory roles in these processes. To evaluate the responses of *SmNBS-LRR* genes to hormones and stress, this study performed a comprehensive expression analysis of all 196 *NBS-LRR* genes in *S. miltiorrhiza* using transcriptome data obtained under various stress conditions (e.g., UV-B, high light stress) and plant hormone treatments (e.g., MeJA, salicylic acid). By comparing the expression patterns of *NBS* genes under different treatment conditions (Figure 5), this study aims to identify key *NBS* genes specifically responsive to stress or hormone induction and further explore their potential roles in plant immune responses and secondary metabolism regulation.

Tanshinones accumulate primarily in the underground roots of *S. miltiorrhiza*. To investigate the association between *NBS* resistance genes and tanshinone biosynthesis, we selected *NBS* genes with high expression levels in roots from the 196 identified *NBS* genes (Figure S2). Given that *NBS-LRR* genes expression is often regulated by MeJA and SA signaling pathways [34], we further screened genes highly expressed across three transcriptome datasets and performed comparative analysis using Venn diagrams (Figures S3 and S4). Additionally, the expression levels of key enzymes involved in *S. miltiorrhiza* secondary metabolite biosynthetic pathways were included to characterize their expression patterns. The analysis results showed that 21 *NBS* genes in *S. miltiorrhiza* roots exhibited significantly increased expression following SA treatment, with most reaching peak expression at 2 h post-induction. Among these, 14 genes belong to the typical CNL subfamily and possess complete N-terminal and LRR domains (*SmNBS7/29/30/35/49/51/58/83/88/91/128/137/148/187*) (Figure 5A). Following MeJA treatment, six NBS genes showed significantly upregulated expression, including SmNBS8/41/21/154, which belong to the CNL subfamily with complete domains. Additionally, the terpene synthase CPS1 and CYP71D375, which are involved in the tanshinone biosynthesis pathway, were also significantly upregulated, reaching peak expression at 1 h post-treatment (Figure 5A). On the other hand, Venn diagram analysis revealed five *NBS* genes whose expression was significantly upregulated simultaneously in roots and across the three hormone treatment transcriptome datasets. Among these, *SmNBS167* belongs to the RNL subfamily, while *SmNBS79/87/152* are members of the CNL subfamily. Concurrently, the key tanshinone biosynthetic enzyme CYP76AH1, CYP76AH3, CYP76AK1, and the upstream diterpene synthase KSL1, also exhibited significantly increased expression levels with similar patterns (Figure 5A). Multiple studies have demonstrated that both MeJA and SA treatments can induce the upregulation of certain NBS-LRR genes. The observed expression changes in NBS genes under hormone treatments further suggest their potential roles in cross-signal integration and regulation within plant immune networks.

Other abiotic stresses, such as UV-B and high light exposure, can also induce transcriptional changes in certain *NBS-LRR* genes. Therefore, we first selected NBS genes highly expressed in the aerial leaf tissues of *S. miltiorrhiza* and performed comparative analyses with *NBS* family genes showing significant expression changes under UV-B and high light stress treatments (Figures S2, S3 and S5). The results showed that 20 *NBS* genes in leaves exhibited increased expression under high light stress, among which 10 of them belong to the typical CNL subfamily with complete domains (*SmNBS35/41/49/51/70/128/189/190/191/192*); most of these genes reached peak expression at 0.5 h post-stress treatment (Figure 5B). Meanwhile, two CYP98A family genes involved in the phenolic acid biosynthesis pathway in *S. miltiorrhiza* also showed significantly increased expression at 2 h after high light stress. Following UV-B stress treatment, 13 NBS genes in leaves exhibited increased expression, among which 6 genes possessed complete domains and belonged to the CNL subfamily (*SmNBS21/56/109/148/154/174*), and the rosmarinic acid synthase (RAS) gene involved in the phenolic acid biosynthesis pathway displayed a similar expression pattern. The Venn diagram shows that eight differentially expressed NBS genes were commonly identified across all three transcriptome datasets, with six of them belonging to the typical CNL subfamily (SmNBS29/30/47/110/157/196) (Figure 5B).

In the above expression pattern analysis, 32 differentially expressed *NBS* genes were identified in the underground root tissues of *S. miltiorrhiza*, while 40 differentially expressed genes were found in the aerial leaf tissues. Comparison revealed only 15 genes were common between these two tissue types, indicating distinct distribution patterns of the *NBS-LRR* gene family in underground versus aerial tissues of *S. miltiorrhiza*, suggesting they may perform different functions. This study contributes to revealing the functional diversity of NBS-LRR resistance proteins in medicinal plants and provides valuable insights for mining functionally important NBS proteins in *S. miltiorrhiza*.

## 3. Discussion

*NBS-LRR* genes originated from the common ancestor of all green plants [35], with the differentiation of the TNL, CNL, and RNL subfamilies occurring prior to the divergence of green algae. The NBS gene family exhibits varying patterns of expansion and contraction across angiosperms. Both the Fabaceae and Rosaceae families show significant expansions [36,37], while the wheat genome contains over 2000 *NBS* genes [38]; however, the Orchidaceae family exhibits a contraction pattern, with the smallest number of NBS genes only five found in Gastrodia elata [39], while the Poaceae family shows a more complex pattern, with *NBS* gene copy number variations reaching up to 66-fold among different species [28,40]. It is hypothesized that such dramatic variation in NBS gene numbers may result from periods of rapid expansion or contraction within the NBS gene family.

This study identified 196 NBS-LRR proteins in the *S. miltiorrhiza* genome (Table S1). By comparing the number and distribution of *NBS-LRR* subfamily genes in other Salvia species, we found that the expansion of the CNL subfamily is widespread within the genus, constituting the vast majority of the *NBS* gene family; it is speculated that in *S. miltiorrhiza* and other Salvia species, CNL subfamily proteins may assemble into complexes that form Ca^2+^ channels, thereby activating plant immune responses [41,42,43]. On the other hand, the NBS proteins in *S. miltiorrhiza* exhibit domain degeneration, with only 31.6% of SmNBS-LRR proteins containing complete domains, and the reduction or loss of TNL and RNL proteins was observed across Salvia species (Figure 2). The observed loss of TNL subfamily may result from the absence of specific signaling components, including NRG1 and SAG101, which has been reported in monocots as well as in certain dicots [28,44]. Most TNL proteins rely on RNL proteins to transduce immune signals [45]; therefore, TNLs and RNLs are frequently lost together [46]. This phenomenon has also been reported in the order Lamiales [47]. Interestingly, the degeneration of subfamilies or domains does not imply a weaker immune capacity; when the environment lacks relevant pathogens, retaining certain NBS genes may impose a fitness cost on plants [48,49]. For example, in environments where pathogen pressure is drastically reduced, such as aquatic environments, TNLs tend to be rapidly lost [10]. Meanwhile, the CC domain of CNLs is not indispensable for the activation of HR; in some NLR genes, the N-terminal region has not fully degenerated and still retains amino acid motifs capable of triggering immune responses in plants [50].

Plant *NBS-LRR* genes play a central role not only in recognizing pathogens and activating immune responses but also in regulating secondary metabolic pathways involved in the synthesis of defense-related metabolites. Activation of the plant defense system induces metabolic pathways that produce common chemical defense compounds, known as phytoalexins [51]. Studies have shown that NBS-LRR-mediated immune responses can upregulate the expression of biosynthetic genes for secondary metabolites such as flavonoids, alkaloids, and terpenoids, thereby enhancing plant resistance to pathogens. This linkage between immunity and metabolism helps plants rapidly establish a multi-layered defense system when subjected to stress. SA- and MeJA-mediated signaling pathways play crucial roles in plant defense against pathogens, leading to increased endogenous levels of SA and MeJA hormones [52,53]; they also play important regulatory roles in the hypersensitive response (HR) against various pathogens. On the one hand, exogenous application of these plant hormones can promote the accumulation of active secondary metabolites such as tanshinones and phenolic acids in *S. miltiorrhiza* [54]. The results showed that, following MeJA treatment, four members of the CNL subfamily (*SmNBS8/41/21/154*) exhibited expression patterns consistent with those of the terpene synthase enzyme CPS1 and the cytochrome P450 enzyme CYP71D375 involved in the tanshinone biosynthetic pathway. On the other hand, SA treatment markedly increased the expression levels of several NLR family genes, among which five members were highly expressed in roots (*SmNBS167* from the RNL subfamily and *SmNBS79* from the CNL subfamily). Notably, a similar expression pattern was also observed for key enzymes in the tanshinone biosynthetic pathway, including CYP76AH1, CYP76AH3, CYP76AK1, and the upstream ent-kaurene synthase SmKSL (miltiradiene synthase), whose transcript levels were likewise significantly elevated. Numerous studies have demonstrated that both MeJA and SA treatments can induce the upregulation of certain *NBS-LRR* genes, suggesting that these *NBS* genes may play important roles in cross-signaling integration and immune network regulation. In light of their coordinated expression with key enzyme genes in the tanshinone biosynthetic pathway, it is speculated that the immune responses mediated by these NLRs may be involved in the regulation of tanshinone metabolism in *S. miltiorrhiza*.

Under high light and UV-B stress, plants experience the accumulation of ROS, DNA damage, and photosystem instability [55], which in turn activate the expression of various defense-related genes and significantly influence the biosynthesis of multiple secondary metabolites, including phenolic acids, flavonoids, and terpenoids. However, reports on the relationship between *NBS-LRR* genes and light responses in plants are relatively limited. The TNL proteins can function as NAD^+^ hydrolases, generating nucleotide-based signaling molecules that activate immune responses [56,57], and plants activate defense responses through light-induced increases in intracellular Ca^2+^ concentrations [58]. Additionally, Rahman et al. [59] used optogenetic tools to demonstrate that light-gated channelrhodopsins can alter physiological responses in tobacco, enhancing defense responses by modulating Ca^2+^ and ROS signaling. Under high-light stress, a total of 20 NBS genes in *S. miltiorrhiza* leaves showed markedly increased expression levels (Figure 5B). Among them, the expression patterns of *SmNBS25*, *SmNBS89*, and *SmNBS189* were consistent with those of the key enzyme CYP98A14 and CYP98A75 in the salvianolic acid biosynthetic pathway [60].

## 4. Materials and Methods

### 4.1. Identification and Classification of SmNBS-LRR

The whole genome of *S. miltiorrhiza* (inbred line bh2-7) was retrieved from IMP database (Integrated Medicinal Plantomics) [61]. To identify the *NBS-LRR* resistance gene families, we downloaded the hidden Markov Model profile (HMM) of the NB-ARC domain (Pfam:PF00931); all proteins were scanned using hmmsearch tool (e-value: 1 × 10^−5^), and those identified as containing an NBS conserved domain were considered as NBS-LRR proteins. We used the CD-HIT command-line tool to eliminate redundant sequences. To classify the subfamilies, we performed domain prediction on the candidate proteins using the Pfam37.4—A database to identify all conserved domains within the sequences.

### 4.2. Phylogenetic Analysis and Classification of NBS-LRR

Phylogenetic analysis was conducted using IQ-TREE 2.0 software [62] to construct a maximum likelihood (ML) tree based on aligned amino acid sequences. The LG substitution model was applied (-m LG), and node support was assessed using 1000 ultrafast bootstrap replicates (-bb 1000) and 1000 SH-aLRT replicates (-alrt 1000). The number of computational threads was automatically determined (-nt AUTO). The resulting phylogenetic tree was visualized using iTOL (https://itol.embl.de/ (accessed on 1 September 2025)) [63].

### 4.3. Gene Structure, Conserved Motif and cis-Regulatory Elements Analysis

The exon–intron distribution data of *S. miltiorrhiza* NBS genes were obtained from the GFF annotation file and visualized using TBtools II (2.311 version) [64]. Conserved motif analysis was performed using the online tool MEME (https://meme-suite.org/meme/tools/meme (accessed on 1 September 2025)) [65]. Conserved domain information of the gene family was analyzed using the Batch CD-Search Tool of NCBI. Promoter sequences 2000 bp upstream of the transcription start sites of *NBS-LRR* genes were extracted using TBtools and submitted to the online PlantCARE database for identification [66] and quantification of cis-acting elements in the promoter regions.

### 4.4. Expression Profiling of NBS-LRR Across Different Tissues and Different Stress Based on RNA-Seq Data

Transcriptome expression data for various tissues of *S. miltiorrhiza* and MeJA-treated samples were downloaded from the IMP (Integrated Medicinal Plantomics) database. Additionally, raw RNA-seq data for SA treatment (PRJNA301529), UV-B stress (PRJNA554403), and light stress (PRJNA1050884) were retrieved from NCBI. These raw SRR files were quantified using Salmon command [67], followed by normalization with the DESeq2 package in R (version 4.5.0). Heatmaps were generated using TBtools, and network Venn diagrams were created using OmicShare tools (https://www.omicshare.com/tools, accessed on 1 September 2025) [68].

## 5. Conclusions

This study systematically mined the *NBS-LRR* resistance gene family in *S. miltiorrhiza* and analyzed subfamily evolution by constructing a phylogenetic tree using predicted NBS-LRR proteins from multiple species. The analysis revealed that Salvia species, including *S. miltiorrhiza*, exhibit contraction and degeneration of the TNL and RNL subfamilies. Through gene domain and conserved motif analysis, as well as cis-acting element prediction and annotation, it was found that *SmNBS-LRR* genes are involved in multiple signaling pathways related to plant hormones and stress responses. Gene expression pattern analysis also revealed differential distribution of *SmNBS-LRR* genes between aerial and underground tissues of *S. miltiorrhiza*, and predicted that multiple *SmNBS-LRR* genes may play regulatory roles in the biosynthesis of secondary metabolites in S. miltiorrhiza. Our study further reveals the association among the *NBS-LRR* gene family, plant hormones, stress responses, and secondary metabolism in *S. miltiorrhiza*. In summary, this research systematically analyzed the *NBS-LRR* genes in S. miltiorrhiza, providing important theoretical foundations for elucidating their functional mechanisms in plant immunity and their applications in breeding.

## Data Availability

The data underlying this article are available in this paper. The datasets in this article were derived from the National Center for Biotechnology Information (NCBI) and the IMP database (Integrated Medicinal Plantomics, https://www.bic.ac.cn/IMP/#/ (accessed on 1 September 2025)).

## Supplementary Materials

The following supporting information can be downloaded at https://www.mdpi.com/article/10.3390/ijms26189063/s1.
