# Supplementary material for: Genome-Wide Characterization and Expression Pattern Analysis Insights into Plant NBS-LRR Gene Family of Salvia miltiorrhiza
Source: Int J Mol Sci. 2025 Sep 17;26(18):9063. doi: 10.3390/ijms26189063 (PMC12469828; doi:10.3390/ijms26189063)
Supplement: Supplementary file 1 [file ijms-26-09063-s001.zip › Supplementary Figure.pdf]

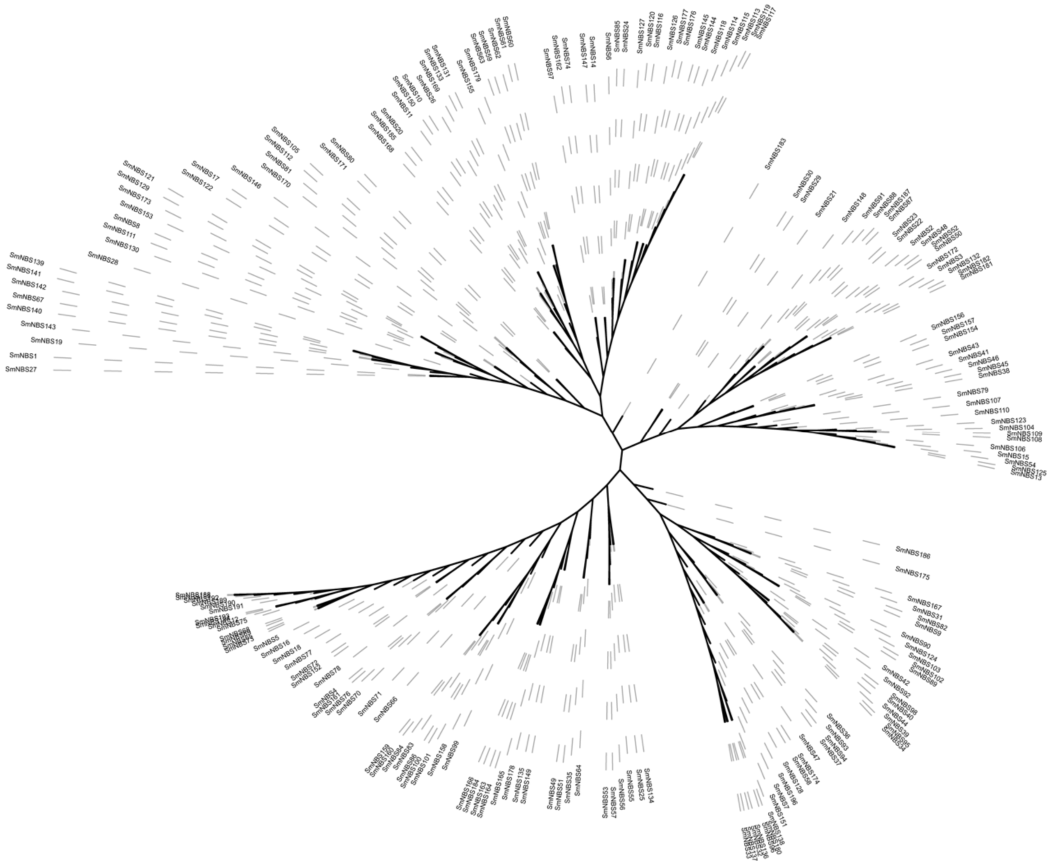

Figure S1. Phylogenetic analysis of the 196 SmNBS-LRRs protein. A maximum likelihood (ML) tree was constructed using IQ-Tree tool.

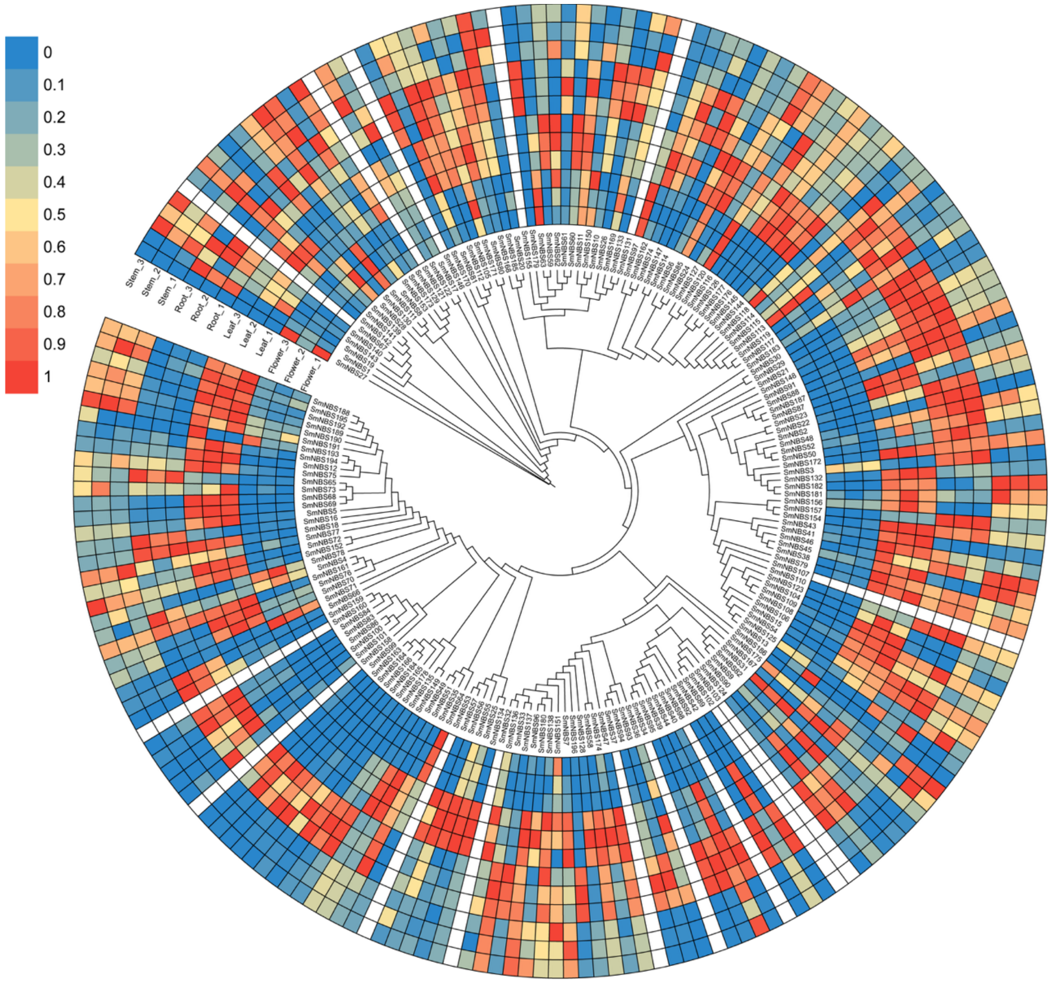

Figure S2. The expression profile of the 196 SmNBS-LRRs involved in 4 tissue (leaf, root, stem, flower).

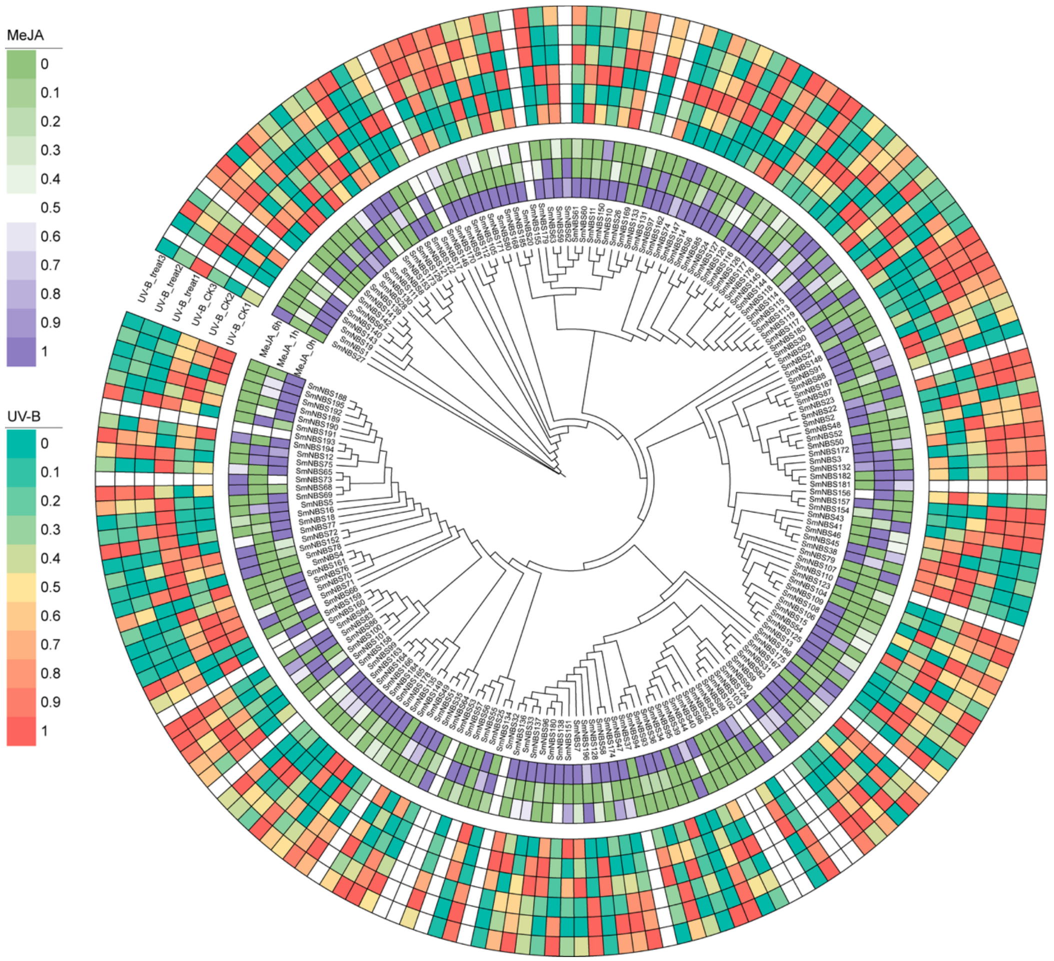

Figure S3. The expression profile of the 196 SmNBS-LRR treated by MeJA (0h, 1h, 6h) and UV-B.

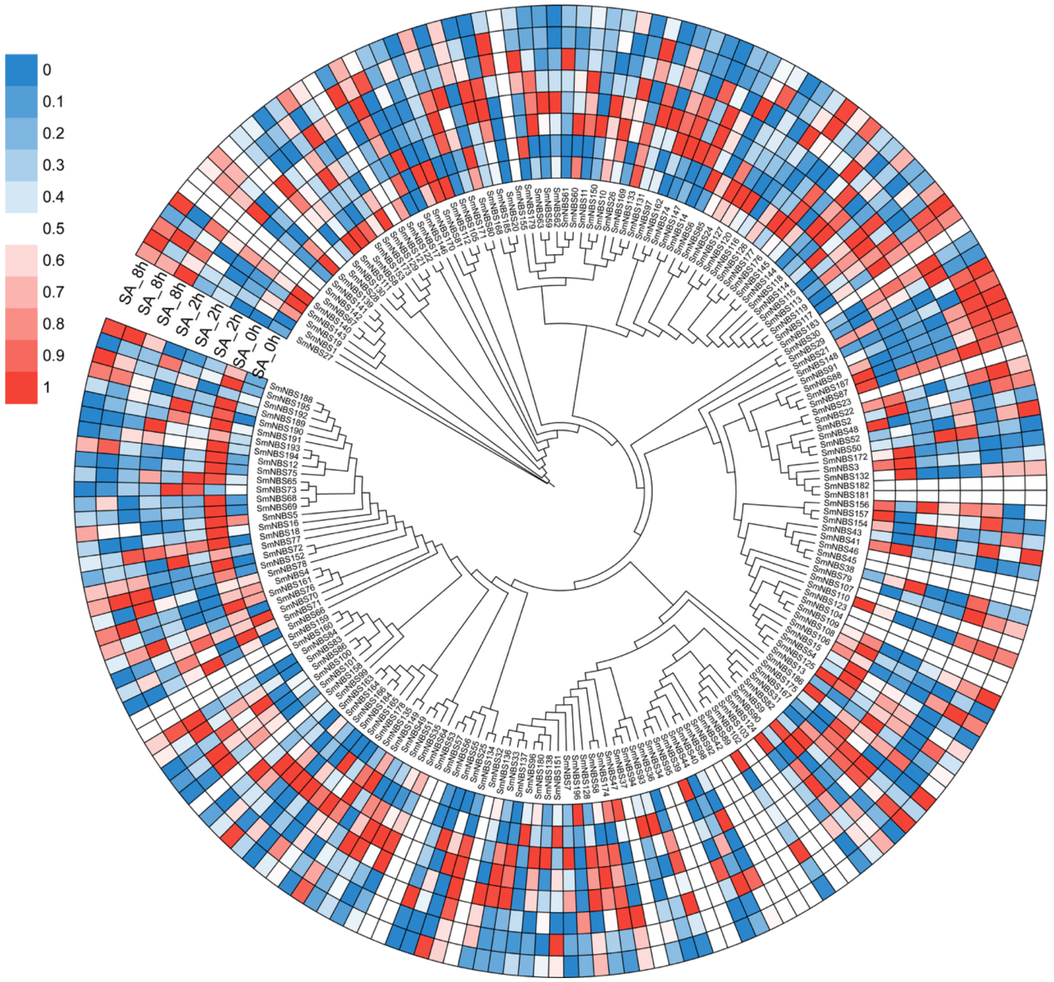

Figure S4. The expression profile of the 196 SmNBS-LRR treated by Salicylic acid (0h, 2h, 8h).

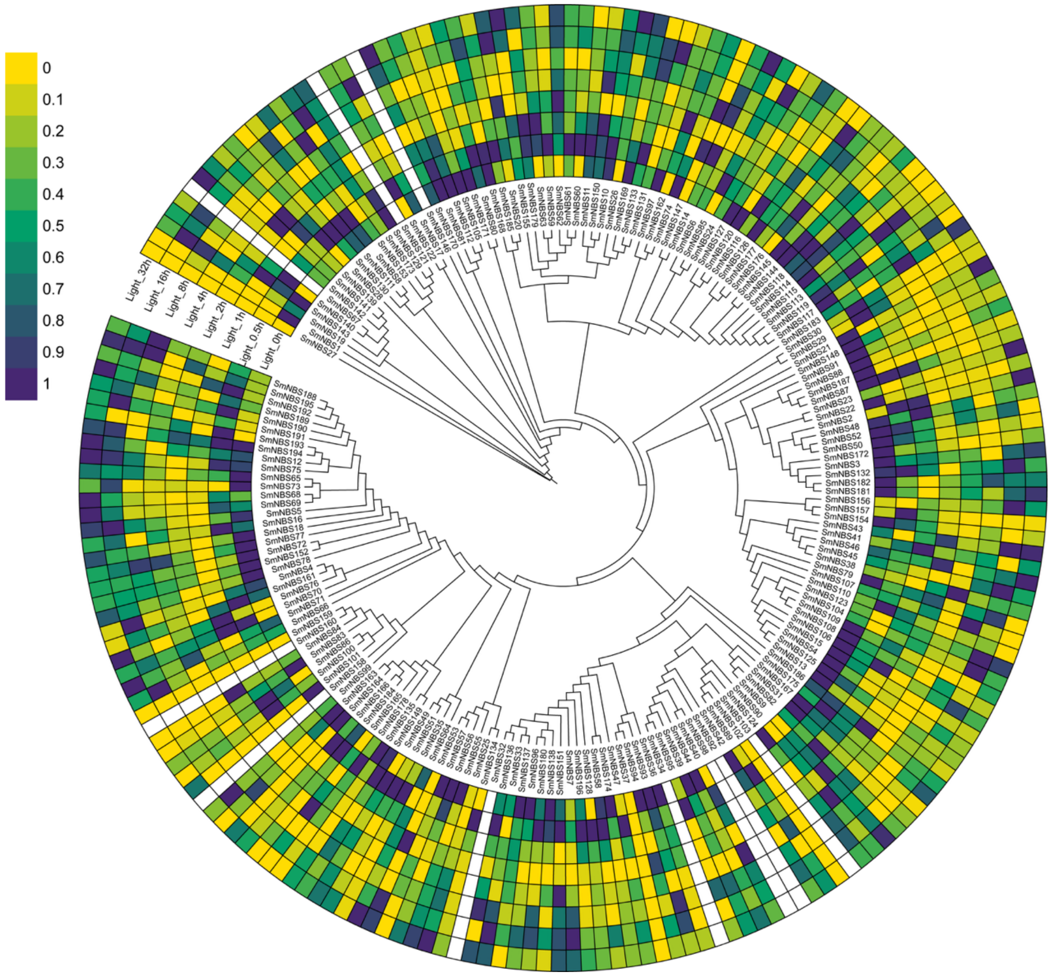

Figure S5. The expression profile of the 196 SmNBS-LRR treated by Light (0h, 0.5h, 1h, 2h, 4h, 8h, 16h, 32h).
